# Multi-criteria decision analysis as an innovative approach to managing zoonoses: results from a study on Lyme disease in Canada

**DOI:** 10.1186/1471-2458-13-897

**Published:** 2013-09-30

**Authors:** Cécile Aenishaenslin, Valérie Hongoh, Hassane Djibrilla Cissé, Anne Gatewood Hoen, Karim Samoura, Pascal Michel, Jean-Philippe Waaub, Denise Bélanger

**Affiliations:** 1 Groupe de Recherche en Épidémiologie des Zoonoses et Santé Publique (GREZOSP), Pavillon de la santé publique, Faculté de médecine vétérinaire, Université de Montréal, Saint-Hyacinthe, CP 5000, Québec, Canada; 2 Département de Géographie, Université du Québec à Montréal, Succursale Centre-ville, Montréal, CP 8888, Québec, Canada; 3 Department of Community and Family Medicine, Geisel School of Medicine, One Medical Center Drive, Lebanon HB 7927, New Hampshire, USA; 4 Laboratory for Foodborne Zoonoses, Public Health Agency of Canada, Saint-Hyacinthe CP 5000, Québec, Canada

**Keywords:** Lyme disease, Lyme borreliosis, Multi-criteria decision analysis, Lyme disease prevention, Vector-borne diseases, Zoonoses, Public health decision-making

## Abstract

**Background:**

Zoonoses are a growing international threat interacting at the human-animal-environment interface and call for transdisciplinary and multi-sectoral approaches in order to achieve effective disease management. The recent emergence of Lyme disease in Quebec, Canada is a good example of a complex health issue for which the public health sector must find protective interventions. Traditional preventive and control interventions can have important environmental, social and economic impacts and as a result, decision-making requires a systems approach capable of integrating these multiple aspects of interventions. This paper presents the results from a study of a multi-criteria decision analysis (MCDA) approach for the management of Lyme disease in Quebec, Canada. MCDA methods allow a comparison of interventions or alternatives based on multiple criteria.

**Methods:**

MCDA models were developed to assess various prevention and control decision criteria pertinent to a comprehensive management of Lyme disease: a first model was developed for surveillance interventions and a second was developed for control interventions. Multi-criteria analyses were conducted under two epidemiological scenarios: a disease emergence scenario and an epidemic scenario.

**Results:**

In general, we observed a good level of agreement between stakeholders. For the surveillance model, the three preferred interventions were: active surveillance of vectors by flagging or dragging, active surveillance of vectors by trapping of small rodents and passive surveillance of vectors of human origin. For the control interventions model, basic preventive communications, human vaccination and small scale landscaping were the three preferred interventions. Scenarios were found to only have a small effect on the group ranking of interventions in the control model.

**Conclusions:**

MCDA was used to structure key decision criteria and capture the complexity of Lyme disease management. This facilitated the identification of gaps in the scientific literature and enabled a clear identification of complementary interventions that could be used to improve the relevance and acceptability of proposed prevention and control strategy. Overall, MCDA presents itself as an interesting systematic approach for public health planning and zoonoses management with a “One Health” perspective.

## Background

Zoonoses, and more generally infectious diseases arising from the interaction of human populations with animals and the environment, are a growing international public health threat that is likely to increase with ongoing globalisation and climate change. Currently, these diseases represent three quarters of all recognized emerging infectious diseases [1]. Recent outbreaks of avian influenza and SARS had expensive and multi-sectoral consequences across continents [2,3]. The worldwide emergence of bovine spongiform encephalopathy and West Nile virus (WNv) in North America are two additional examples of such diseases that have caused widespread concern with the general public and decision makers [4,5].

Interacting at the human-animal-environment interface, zoonoses that involve vectors and wildlife species have particular characteristics making them difficult to prevent and control. On the one hand, they involve multiple species, often with complex ecologies, making control or eradication very difficult. On the other hand, because of this ecological complexity, preventive and control interventions of zoonoses can have environmental, social and economic impacts. For example, larvicides used in attempts to control WNv during the 1999–2007 outbreak in Canada were found to be toxic for some wild bird and fish species, and their use was publicly criticized by local media and experts [6]. Moreover, zoonoses pose important prevention and control challenges because multiple organisations and stakeholders share responsibilities with regards to public health actions; however, the implementation and anticipated effects of cross-sectoral interventions can be more difficult to predict for strategic planners and decision-makers. This complexity calls for transdisciplinary and multi-sectoral approaches in order to achieve effective disease management, a call which can no longer be ignored [7].

A “One Health” approach is needed to develop effective management for zoonoses. The “One Health” approach recognizes the intimate linkages between human, animal and environmental health systems and proposes an international, interdisciplinary, and cross-sectoral approach to disease surveillance, monitoring, prevention, control and mitigation of emerging and re-emerging diseases [8]. In recent years, this approach has been adopted by several national and international organisations as a promising way to improve public health interventions [9,10].

Multi-criteria decision analysis (MCDA) methods come from the field of operations research and are commonly used in environmental, industrial and business management [11,12]. Multiple MCDA algorithms exist to analyse different types of decision problems, one of which consists of ranking and comparing alternatives based on multiple criteria that can be evaluated using either quantitative and/or qualitative indicators [13]. In the absence of quantitative data for a criterion in a specific context, MCDA methods allow for the incorporation of qualitative evaluations, for example based on expert opinion. Moreover, it offers the possibility, if needed, for a participatory approach with stakeholders concerned by a particular issue, allowing them to actively engage in all stages of the decision analysis supported by MCDA. As such, MCDA methods are well suited for complex, transdisciplinary and multi-sectoral decision-making problems such as zoonotic disease management [14,15]. The use of MCDA in public health has thus far been limited but is emerging as a complementary method for evidence-based public health [16-18]. It has been used in different contexts, such as for the prioritization of general health issues, including zoonoses [17-23], and to compare potential alternatives or interventions for public health management. For this later goal, most published studies have been in the field of environmental health, but some more recent studies were done to prioritize interventions for infectious diseases management [24-26]. Fewer studies have attempted to confront zoonotic or animal related disease problems with an MCDA approach: one such study used MCDA to compare strategies for the elimination of carcasses following a hypothetical event of food-industry targeted terrorism [27], another used MCDA to compare different methods of quarantine and control during animal disease epidemics [28,29]. Hongoh and colleagues [30] proposed a general model and discussed the potential strengths of the use of MCDA methods for vector-borne disease management with explicit spatial considerations.

Lyme disease, a zoonosis caused by the bacteria *Borrelia burgdorferi* and transmitted to humans via the bites of ticks infected from animal reservoirs, is one of the most common vector-borne diseases in temperate countries and is a good example of a public health issue at the human-animal-environment interface [31]. The incidence of Lyme disease has been increasing annually in North America, particularly in the north-eastern United States where more than 20,000 cases are reported annually [31]. In the Canadian province of Quebec, the incidence of Lyme disease has thus far been low (0.5 per 100 000 in 2011 according to a recent report by the Quebec National Institute of Public Health [32] compared to rates of 60.3, 67.3 and 76 per 100 000 in the same year in the neighbouring states of Maine, New Hampshire and Vermont respectively [33]), but could grow to 8 000 cases annually by 2050 in the southeast and the central southern parts of Canada [34-37]. The first autochtonous human case of Lyme disease in Quebec was reported in 2008 (Quebec National Institute of Public Health, unpublished observations). Black legged tick populations, the vector of *B. burgdorferi*, are now recognized as having become established in the southern part of the province, with up to 13% of ticks infected with *B. burgdorferi*[37]. Given these changing dynamics, the Quebec public health authorities have expressed the desire to proactively respond to this public health concern and to develop a Lyme disease management plan. As such, this is an ideal context for the use of MCDA methods for zoonotic disease management. In the Quebec health system, public health responsibilities, including Lyme disease prevention, are shared between provincial and regional public authorities. The Ministry of Health and Social Services, supported by the National Institute of Public Health in Quebec, is the provincial authority and first level responsible for applying provincial public health programs in collaboration with regional authorities [38]. Each of the 18 administrative regions of the province has their own Health and Social Services Agency. No province-wide program for Lyme disease management currently exists due to the fact that the disease is presently only concentrated in the southern part of Quebec and has affected only a few regions. This article aims to present the context, goals, methods and results from a study of the use of MCDA for Lyme disease management in Quebec, Canada.

## Methods

The study was conducted between September 2010 and February 2012 with the general objective of identifying, evaluating and ranking different strategies for Lyme disease management in Quebec, in order to support decision-making and program direction by public health authorities. A research team composed of senior and junior researchers active in the fields of public health, veterinary public health and decision analysis research was assembled specifically for this study.

Three main intervention areas were first identified to address Lyme disease management in a comprehensive manner (see the result section for the detailed research questions): preventive communication strategies (COMM), surveillance strategies (SURV) and control strategies (CONT). Preventive communication strategies refers to all modes of communication implemented by public health authorities to prevent Lyme disease in humans; surveillance strategies refers to vector, animal and human related surveillance activities designed to monitor the disease and the state of the infection in the reservoir and the vector; and control strategies refers to field interventions which can be implemented to reduce the risk of transmission of the infectious agent to humans. This article will focus on the results from the MCDA approach applied to the ranking of surveillance and control strategies. Analyses of communication strategies with MCDA will not be presented in the present paper as they required a slightly different methodological framework. The term ‘intervention’ will be use to refer to the specific strategies included in this study for surveillance and control.

**Figure 1:**
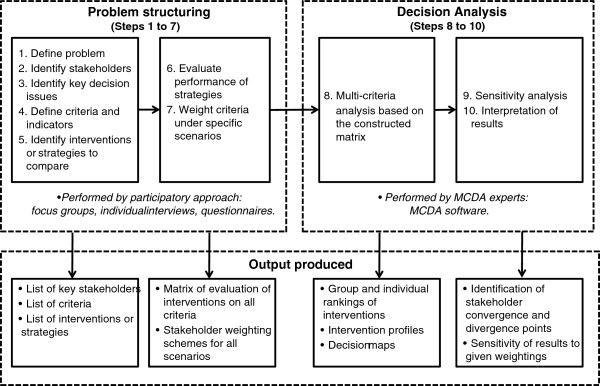
MCDA general steps and outcomes.

The MCDA process used for this study can be divided into ten general steps, each with specific methods (Figure 1). These ten steps are non-linear and may require a few iterations. For example, the identification and inclusion of certain strategies in the model can lead to the inclusion of additional stakeholders in the group as was the case for our project. A participatory approach (focus groups, individual interviews, questionnaires) with a group of stakeholders was adopted for this study. A stakeholder was defined as a person representing an organisation or a group with direct responsibilities or with specific interests in Lyme disease management. This included representatives of governmental or non-governmental organisations, health professionals and experts (different from the research team). Stakeholders were asked to represent the preferences of their organisation and not their own preferences. To make this possible, they were invited to consult other colleagues in their organisation throughout the MCDA process. Stakeholders participated most intensively in the problem definition stage, identification of the extended stakeholders group, identification of key decision issues, translation of these issues into measurable criteria, identification of Lyme disease intervention strategies (steps 1 to 5) and weighting of the criteria (step 7).

The identification of issues (step 3), the definition of criteria (step 4) and the selection of interventions (step 5) were done using an iterative process with the stakeholders: the research group proposed a first set of interventions and criteria along with their definitions, indicators for each criteria and appropriate measurement scales based on their understanding of the major surveillance and control issues for Lyme disease. This initial set was discussed and modified by the stakeholders by means of a focus group discussion. The final versions of the criteria (name, definition, indicator, measurement scales) and interventions lists were then validated by each stakeholder using a web-based questionnaire.

The evaluation of performance parameters for the retained list of interventions was carried out over each criterion (step 6) using literature supported data when available. When data were not available to evaluate parameters for criteria, Delphi surveys were conducted with stakeholders (CONT model) and external experts (SURV model). If there was no consensus on parameters after two iterations of the Delphi process, the mean value was selected. For the CONT model, selected stakeholders and members of the research team were consulted as experts for the determination of parameters. For the SURV model, a group of three external experts in Lyme surveillance were consulted.

Individual weighting of all criteria was done by each stakeholder under two different scenarios (step 7). The first scenario described the current epidemiological situation of Lyme disease in Quebec (referred to as “emergence scenario”). The second scenario described a hypothetical substantial increase in the annual number of human cases of Lyme disease in Quebec, coupled with more intensive media coverage and public awareness (referred to as “epidemic scenario”). Each stakeholder was asked to create model-specific weighting schemes based on the stakeholder’s perceived importance of criteria for decision-making in line with their organisational affiliation. This was done by first distributing 100 points among the criteria categories and then re-distributing the category specific points to all criteria within each category. A tool was created using Excel software version 2007 to facilitate this exercise. Individual support was offered to stakeholders to complete the weighting when needed to ensure a good comprehension of the process.

Decision analysis (steps 8 to 10) was carried out in D-Sight (software version 3.3.2) which uses the PROMETHEE (Preference Ranking Organization Method for Enrichment Evaluations) methods and gives access to the GAIA (Geometrical Analysis for Interactive Aid) visual model to explore analysis results [39-41]. PROMETHEE methods enable fully comparative rankings of a set of alternatives [11]. The decision map, which is called a GAIA plan and is generated by the D-sight software, is a two-dimensional graphical representation of a stakeholder’s position, dictated by their individual preferences (ranking of interventions). The decision map is referenced with a decision axis (in red), which points toward the “best” interventions in accordance with the group scores of interventions on all included criteria. The proximity of stakeholder’s positions within the GAIA plan represents proximity in their overall preferences. This graphical representation of stakeholder values can be used to identify potential coalitions or clusters of positions among stakeholders and to identify positions that are distinctive from that of other stakeholders by their distances in relation to others or to the decision axis. Similarly to positioning stakeholders, the GAIA plan also positions all interventions in relation to the overall decision axis. Multi-criteria analyses carried out in D-sight assess the performance of interventions over all criteria resulting in numerical scores for each intervention. D-sight uses specific algorithms based on a pair wise method of analysis to calculate scores (PROMETHEE I and II) [33]. The intervention scores and weighting schemes set by stakeholders can be combined to produce three sets of results of particular interest in our study: 1) group rankings of the interventions, representing an ordered ranking of most preferred to least preferred intervention that takes all weighting schemes and intervention performance scores into account; 2) individual rankings for each stakeholder, representing the most preferred to least preferred intervention for a particular stakeholder given their specific weighting scheme; and 3) individual performance of interventions for every criterion, showing how an intervention performs on every criterion independently of all stakeholder weighting schemes. Intervention scores represent the relative performance of an intervention with respect to another, and as such scores do not have individual meanings by themselves. The objective of these scores is to provide a common numeric value to enable comparisons of interventions. Further details on MCDA methods have been published by Figueira and colleagues [42].

Analyses presented in this paper include group rankings and individual performance of selected interventions, as well as decision maps of stakeholder’s preferences for particular interventions (GAIA planes), and an example of a sensitivity analysis of one stakeholder’s weighting schemes. Each of the 10 MCDA steps was conducted separately for each intervention area (SURV and CONT). Results for the first 5 steps will be presented using SURV and CONT models and a more in depth illustration will be presented for steps 6 to 10 using the CONT model.

## Results

### Problem definition and identification of stakeholders (Steps 1 & 2)

To address the general problem of identifying, evaluating and ranking different strategies for Lyme disease management in Quebec, five governmental and one academic organisations involved in Lyme disease management were identified and invited to participate in the MCDA process. Eight representative stakeholders agreed to participate. The composition of the group was defined in order to capture the multidisciplinary nature of Lyme management (Table 1). For the examination of surveillance and control strategies, two models were developed to address two specific research questions identified by the stakeholders: 1) what strategies are most effective for Lyme disease surveillance? (SURV model); and 2) what interventions are most effective for the prevention and control of Lyme disease? (CONT model). In the context of this study, effectiveness represents the overall relative performance of strategies and interventions over multiple decision criteria, taking into account their importance as defined by the stakeholders for the Quebec context.

**Table 1:** Composition of the stakeholder group

**Models**	**Organisations (number of stakeholders)**
SURV and CONT	Québec National Institute of Public Health (Institut national de santé publique du Québec): Infectious diseases sector (2), Environmental health sector (1)
	National Public Health Laboratory (Laboratoire national de santé publique) (1)
	Ministry of Agriculture, Fisheries and Food (Ministère de l’agriculture, des pêcheries et de l’alimentation du Québec) (1)
	Ministry of Natural Resources and Wildlife (1)
	Montérégie Regional Board of Health and Social Services (1)
	Academic expert (1)

### Criteria (Steps 3 & 4)

Sixteen criteria were selected for the two models to evaluate the effectiveness of interventions relative to the main issues of Lyme surveillance and control (Table 2). These criteria were divided into five general categories: public health criteria (PHC), animal and environmental health criteria (AEC), social impact criteria (SIC), strategic, economic and operational impact criteria (SEC) and surveillance criteria (SUC). Indicators were defined for each criterion and used qualitative scales for measurement. Categorical ordinal scales were used for all criteria, except for AEC1 and AEC2 where a multiplicative indicator was defined.

**Table 2:** Criteria and measurement scales used in the surveillance (SURV) and control (CONT) models

**Category**	**Criteria**	**Scale**	**Model**	
			**SURV**	**CONT**
Public health criteria (PHC)	PHC1 Reduction in incidence of human cases	0: Nil; 1: Low; 2: Moderate; 3: High		X
	PHC2 Reduction in entomological risk	0: Nil; 1: Low; 2: Moderate; 3: High		X
	PHC3 Impacts of adverse health effects	0: Nil; 1: Indirect effects on mental or social health; 2: Direct effects on physical health		X
Animal and environmental health criteria (AEC)	AEC 1 Impact on habitat	Surface*Sensitivity*Intensity^1^		X
		Surface : 1: Nil; 2: Small scale;		
		3: Large scale; Sensitivity: 1: Nil; 2: Land;		
		3: Water ; 4: Land and water; Intensity: 1: Nil; 2: Fences;		
		3: Mowing; 4: Acaricides; 5: Removal of vegetation or burning		
	AEC 2 Impact on wildlife	Number*Species*Intensity^2^		X
		Number: 1: Nil; 2: Effect on specific species;		
		3: Effect on several species; Species: 1: Nil,		
		2: low valued species; 3: Highly valued species; Intensity: 1: No effect; 2: Morbidity; 3: Mortality		
Social impact criteria (SIC)	SIC 1 Level of public acceptance	1: Nil; 2: Low; 3: Moderate; 4: High		X
	SIC 2 Proportion of population benefitting from intervention	1:<25%; 2:25-50%; 3:50-75%; 4:>75%		X
Strategic, economic and operational impact criteria (SEC)	SEC1 Cost to the public sector	0: Nil; 1: Low; 2: Moderate; 3: High	X	X
	SEC2 Cost to the private sector	0: Nil; 1: Low; 2: Moderate; 3: High	X	X
	SEC3 Delay before results	1: Days; 2: Weeks; 3: Months; 4: Years	X	X
	SEC4 Complexity	1: Simple (minor institutional changes);	X	X
		2:Intermediate (necessitates new hires); 3: Moderate (necessitate new work teams in one sector of intervention); 4: Complex (requires inter-sectoral/inter-institutional changes);		
		5: Very complex (necessitates creation of new structures or organisations)		
	SEC5 Impact on organisation’s credibility	0: Nil; 1: Low; 2: Moderate; 3: High		X
Surveillance criteria (SUC)	SUC1 Detection of zones where tick populations are present	1: Less than 10%; 2: Low (11-50%); 3: Moderate (51-70%); 4: High (>71%)	X	
	SUC2 Identification of zones where tick populations are established	1: Less than 10%; 2: Low (11-50%); 3: Moderate (51-70%); 4: High (>71%)	X	
	SUC3 Identification of Lyme endemic zones	1: Less than10%; 2: Low (11-50%);3: Moderate (51-70%); 4: High (>71%)	X	
	SUC4 Quality of data	1: Poor; 2: Medium; 3: High	X	

^1^The score is calculated using a multiplication of three indicators: surface, sensitivity and intensity.

^2^The score is calculated using a multiplication of three indicators: number, species and intensity.

### Strategies and interventions (Step 5)

For the SURV model, 11 interventions were identified and consisted of various passive and active surveillance strategies targeting vectors, domestic animals and humans: passive surveillance of vectors found on humans (SURV1a) and animals (SURV1b); active surveillance of vectors by flagging or dragging (SURV2a), by trapping of small rodents (SURV2b) and from hunted deer (SURV2c); passive surveillance of domestic animals seropositive cases of *B. burgdorferi* funded by the animal owners (SURV3a), funded by the private sector (industry) (SURV3c) or funded by the public sector (SURV3b); active surveillance of cases of Lyme disease in domestic animals (SURV4); passive surveillance of suspected and confirmed cases of Lyme disease in humans (SURV5); sentinel surveillance of suspected cases in humans (SURV6). For the CONT model, 16 interventions were selected, again with the aim to compare different approaches for Lyme disease prevention and control targeting vectors, vector hosts and human populations. Vector targeted interventions included: small (CONT1a) and large (CONT1b) scale acaricide applications, dessicants/insecticidal soap applications (CONT2), removal of tick habitats by small scale landscaping bordering houses and alleys (CONT3a) or by large scale landscaping in public forested areas (CONT3b). Vector host targeted interventions included: the use of ‘4-poster’ device to control tick infestation on deer (CONT4) or feed-administered ivermectin (CONT5), reduction of deer populations by increased hunting quotas (CONT6a), by culling (CONTb), or by exclusion of deer from public areas (CONT7), tick control on small rodents by ‘Damminix’ device (CONT8) or fipronil application (CONT9). Human population targeted interventions included: exclusion of people from high risk areas (CONT10), vaccination (CONT11), development of Lyme disease clinics to facilitate diagnostic and treatment (CONT12)). All selected CONT interventions have been used in the past to prevent and control Lyme disease in experimental or real life contexts. A ‘status quo’ intervention (CONT0) was also included to represent a ‘no additional measures taken’ option where only a basic public communications strategy for Lyme disease prevention is provided by public health authorities with dissemination via web sites and occasional press releases.

### Evaluation of performance parameters (Step 6)

Each intervention was evaluated on each of the selected criteria using the defined measurement scales and resulting in the creation of two performance matrices, one for each model (Table 3). Performance parameters on criteria varied depending on the intervention. For example, CONT1b (large scale acaricide application) had the highest parameter value (equal to 48) for criterion AEC1 (impact on habitat), meaning that this intervention would have the highest impact on habitat among all interventions included in the matrix. CONT0 (status quo), CONT11 (vaccination) and CONT12 (development of Lyme disease clinics to facilitate diagnostic and treatment) had the lowest parameter values for criterion AEC1 (equal to 1), meaning that these interventions would have the lowest impact on habitat.

**Table 3:** Performance matrices for the surveillance (SURV) and control (CONT) models

	**Performance parameters for each criteria**																
		**PHC**			**AEC**		**SIC**		**SEC**					**SUC**			
	**Intervention**	**PHC1**	**PHC2**	**PHC3**	**AEC1**	**AEC2**	**SIC1**	**SIC2**	**SEC1**	**SEC2**	**SEC3**	**SEC4**	**SEC5**	**SUC1**	**SUC2**	**SUC3**	**SUC4**
CONT model	CONT0	***1***	***0***	***0***	***1***	***1***	**3**	**4**	**1**	**0**	**3**	**1**	**1**	**N/A**			
	CONT1a	***2***	***3***	***2***	***16***	***8***	**2**	**1**	**1**	**1**	***2***	**4**	**3**				
	CONT1b	***2***	***3***	***2***	***48***	***18***	**2**	**4**	**2**	**0**	***2***	**4**	**3**				
	CONT2	***1***	***2***	***2***	***24***	***8***	**2**	**1**	**1**	**0**	***2***	**4**	**2**				
	CONT3a	***2***	***3***	***1***	***20***	***4***	**3**	**1**	**1**	**0**	**2**	**4**	**2**				
	CONT3b	***2***	***3***	***2***	***30***	***9***	**2**	**1**	**1**	**0**	**2**	**4**	**3**				
	CONT4	***2***	***3***	***2***	***3***	***12***	**3**	**1**	**2**	**1**	***4***	**4**	**2**				
	CONT5	***1***	***2***	***2***	***3***	***12***	**3**	**1**	**1**	**1**	***4***	**4**	**3**				
	CONT6a	***0***	***2***	***2***	***3***	***18***	**2**	**2**	**0**	**0**	**4**	**4**	**3**				
	CONT6b	***1***	***2***	***2***	***3***	***27***	**1**	**2**	**2**	**0**	**4**	**4**	**4**				
	CONT7	***1***	***2***	***0***	***12***	***6***	**3**	**1**	**2**	**2**	**4**	**4**	**2**				
	CONT8	***0***	***1***	***2***	***3***	***8***	**3**	**1**	**2**	**1**	**3**	**4**	**2**				
	CONT9	***0***	***1***	***2***	***3***	***8***	**3**	**1**	**2**	**1**	**3**	**4**	**2**				
	CONT10	***1***	***0***	***0***	***3***	***1***	**3**	**3**	**0**	**0**	**2**	**4**	**3**				
	CONT11	***2***	***0***	***2***	***1***	***1***	**3**	**3**	**1**	**0**	***2***	**5**	**2**				
	CONT12	***0***	***0***	***0***	***1***	***1***	**4**	**3**	**1**	**0**	**2**	**5**	**2**				
SURV model	SURV1a	N/A							**2**	**1**	**3**	**1**	**N/A**	**3**	**3**	**3**	**2**
	SURV1b								**2**	**1**	**3**	**1**		**4**	**3**	**4**	**2**
	SURV2a								**3**	**1**	**3**	**2**		**4**	**4**	**3**	**3**
	SURV2b								**3**	**1**	**3**	**4**		**4**	**4**	**3**	**3**
	SURV2c								**3**	**1**	**3**	**4**		**3**	**4**	**3**	**2**
	SURV3a								**2**	**1**	**3**	**4**		**3**	***2***	***2***	**2**
	SURV3b								**1**	**2**	**3**	**4**		**3**	***2***	***2***	**2**
	SURV3c								**2**	**1**	**3**	**4**		**3**	***2***	***2***	**2**
	SURV4								**3**	**1**	**3**	**4**		**3**	**3**	**4**	**3**
	SURV5								**2**	**1**	**3**	**3**		**4**	**4**	**3**	**3**
	SURV6								**2**	**1**	**3**	**3**		**2**	**3**	**3**	**3**

Parameters in bold indicate parameters reviewed by an expert panel (Delphi surveys).

Parameters in bold italics indicate parameters based on literature reviews.

### Weighting of criteria (Step 7)

The weighting of the retained criteria varied in accordance with stakeholder values in each of the models and varied between both scenarios (Table 4). Public health was the category which generally received the highest weighting. For the CONT model, under the epidemic scenario, five stakeholders (S3, S4, S5, S6 and S7) gave additional weight to the public health criteria at the expense of other categories. For example, stakeholder S4 respectively assigned 30, 25, 25 and 20 points to the public health, animal and environmental impacts, social impacts and strategic, economic and operational criteria categories under the emergence scenario, but assigned weightings of 40, 25, 25 and 10 points under the epidemic scenario for the same categories.

**Table 4:** Stakeholder weights (S1 to S8) under the emergence (EM) and the epidemic scenario (EP) for the surveillance (SURV) and control (CONT) models

**Criteria**		**Weights**															
		**SURV model**															
		**S1**		**S2**		**S3**		**S4**		**S5**		**S6**		**S7**		**S8**	
		**EM**	**EP**	**EM**	**EP**	**EM**	**EP**	**EM**	**EP**	**EM**	**EP**	**EM**	**EP**	**EM**	**EP**	**EM**	**EP**
SUC	SUC1	13	13	13	13	21	21	20	21	12	18	25	23	2	3	7	8
	SUC2	13	13	13	13	14	0	20	21	12	18	8	11	8	12	21	16
	SUC3	13	13	15	15	14	18	16	6	12	18	4	17	25	38	21	32
	SUC4	13	13	10	10	21	21	24	12	24	18	26	11	5	3	21	24
	**Total**	**50**	**50**	**50**	**50**	**70**	**60**	**80**	**60**	**60**	**70**	**63**	**63**	**40**	**60**	**70**	**80**
SEC	SEC1	9	9	15	15	9	8	6	8	10	6	3	2	20	13	6	6
	SEC2	9	9	15	15	0	0	2	2	10	6	10	10	20	13	6	4
	SEC3	13	13	15	15	9	16	6	16	10	12	10	10	10	7	6	6
	SEC4	19	19	5	5	12	16	6	14	10	6	15	15	10	7	12	4
	**Total**	**50**	**50**	**50**	**50**	**30**	**40**	**20**	**40**	**40**	**30**	**37**	**37**	**60**	**40**	**30**	**20**
Total		100															
Criteria		CONT model															
		S1		S2		S3		S4		S5		S6		S7		S8	
		EM	EP	EM	EP	EM	EP	EM	EP	EM	EP	EM	EP	EM	EP	EM	EP
PHC	PHC1	8	8	21	21	20	25	12	20	16	25	10	4	25	35	30	30
	PHC2	8	8	3	3	8	13	9	8	12	13	3	6	5	5	10	10
	PHC3	8	8	6	6	12	13	9	12	12	13	8	16	10	10	10	10
	**Total**	**25**	**25**	**30**	**30**	**40**	**50**	**30**	**40**	**40**	**50**	**21**	**26**	**40**	**50**	**50**	**50**
AEC	AEC 1	13	13	15	10	5	3	13	13	5	5	13	14	15	15	10	10
	AEC 2	13	13	15	10	5	3	13	13	5	5	13	14	10	10	10	10
	**Total**	**25**	**25**	**30**	**20**	**10**	**5**	**25**	**25**	**10**	**10**	**26**	**29**	**25**	**25**	**20**	**20**
SIC	SIC 1	18	18	5	10	10	4	15	10	13	10	4	4	10	10	11	9
	SIC 2	8	8	5	10	10	6	10	15	13	10	4	9	5	5	5	6
	**Total**	**25**	**25**	**10**	**20**	**20**	**10**	**25**	**25**	**25**	**20**	**8**	**13**	**15**	**15**	**15**	**15**
SEC	SEC1	3	3	8	8	5	4	6	2	5	3	15	10	6	3	3	2
	SEC2	3	3	8	8	0	0	2	1	3	2	15	5	6	3	3	2
	SEC3	5	5	6	6	6	11	4	3	5	6	1	8	3	1	2	3
	SEC4	9	9	3	3	11	11	4	2	5	4	8	4	2	1	4	3
	SEC5	5	5	6	6	9	11	4	2	8	5	6	5	3	2	4	5
	**Total**	**25**	**25**	**30**	**30**	**30**	**35**	**20**	**10**	**25**	**20**	**45**	**32**	**20**	**10**	**15**	**15**
Total		100															

Bold font indicates the weight totals of criteria within each category.

### Decision analysis (Step 8 to 10)

Group rankings of interventions were performed for both models using the individual stakeholder’s values expressed via criteria weightings (Table 5). Group scores ranged from −0.34 to 0.45 for the SURV model and from −0.33 to 0.43 for the CONT model. For both models, the three preferred interventions were the same regardless of the epidemiological scenario (emergence or epidemic). For the SURV model, the three preferred interventions were SURV2a (active surveillance of vectors by flagging or dragging), SURV2b (active surveillance of vectors by trapping of small rodents) and SURV1a (passive surveillance of vectors of human origin). For the CONT model, CONT0 (status quo – basic preventive communications), CONT11 (human vaccination) and CONT3a (small scale landscaping) were the three preferred interventions. CONT6b (deer culling) and CONT6a (deer hunting) were classified as the least preferable interventions under both scenarios.

**Table 5:** Group ranking of interventions for the surveillance (SURV) and control (CONT) models

**SURV model**	**Emergence**		**Epidemic**	
	**Rank**	**Score**	**Rank**	**Score**
SURV2a – Active surveillance of vectors *I. scapularis* (flagging or dragging)	1	0.43	1	0.45
SURV2b – Active surveillance of vectors *I. scapularis* (trapping of small rodents)	2	0.40	2	0.42
SURV1a – Passive surveillance of vectors *I. scapularis* originating from humans	3	0.07	3	0.10
SURV6 – Sentinel surveillance of suspected Lyme cases in humans	4	0.03	5	−0.01
SURV1b – Passive surveillance of vectors *I. scapularis* originating from animals	5	0.00	4	0.04
SURV5 – Passive surveillance of human Lyme disease cases	6	−0.08	9	−0.14
SURV2c – Active surveillance of vectors *I. scapularis* (from hunted deer)	7	−0.13	7	−0.17
SURV3a – Passive surveillance of seropositivity to *B. burgdorferi* in domestic animals, funding of tests supported by animal owners	8	−0.14	6	−0.12
SURV3c – Passive surveillance of seropositivity to *B. burgdorferi* in domestic animals, funding of tests supported by the public sector	8	−0.14	6	−0.12
SURV3b – Passive surveillance of seropositivity to B *.burgdorferi* in domestic animals, funding of tests supported by the private sector (industry)	9	−0.17	9	−0.14
SURV4 – Active surveillance of domestic animal cases of Lyme disease	10	−0.27	8	−0.34
**CONT model**	Rank	Score	Rank	Score
CONT0 – Status quo (basic preventive communication strategy)	1	0.43	1	0.39
CONT11 – Human vaccination^1^	2	0.31	2	0.31
CONT3a – Small scale landscaping (removal of tick habitats)	3	0.28	3	0.3
CONT10 – Excluding people from high-risk public areas	5	0.25	4	0.29
CONT12 – Making available special Lyme disease diagnostic/treatment clinic(s)	6	0.23	5	0.2
CONT4 – ‘4-poster’ device[47]	8	0.03	6	0.06
CONT7 – Exclusion of deer by fencing	9	−0.04	9	−0.02
CONT1a – Small scale acaricide application to kill free-living ticks	9	−0.04	7	0.01
CONT3b – Large scale Landscaping (removal of tick habitats)	10	−0.07	10	−0.03
CONT1b – Large scale acaricide application to kill free-living ticks	11	−0.08	8	−0.01
CONT2 – Application of desiccants/insecticidal soap	12	−0.14	11	−0.15
CONT5 – Feed-administered ivermectin to deer at bait stations to control ticks	13	−0.15	12	−0.17
CONT8 – ‘Damminix’ device[48]	14	−0.22	13	−0.25
CONT9 – Bait boxes to deliver a passive application of fipronil to rodents[49]	14	−0.22	13	−0.25
CONT6a – Deer hunting	15	−0.25	14	−0.29
CONT6b – Deer culling	16	−0.33	15	−0.31

^1^ Currently, no licensed vaccine exists for human use. Data used for this analysis come from [50]

**Figure 2:**
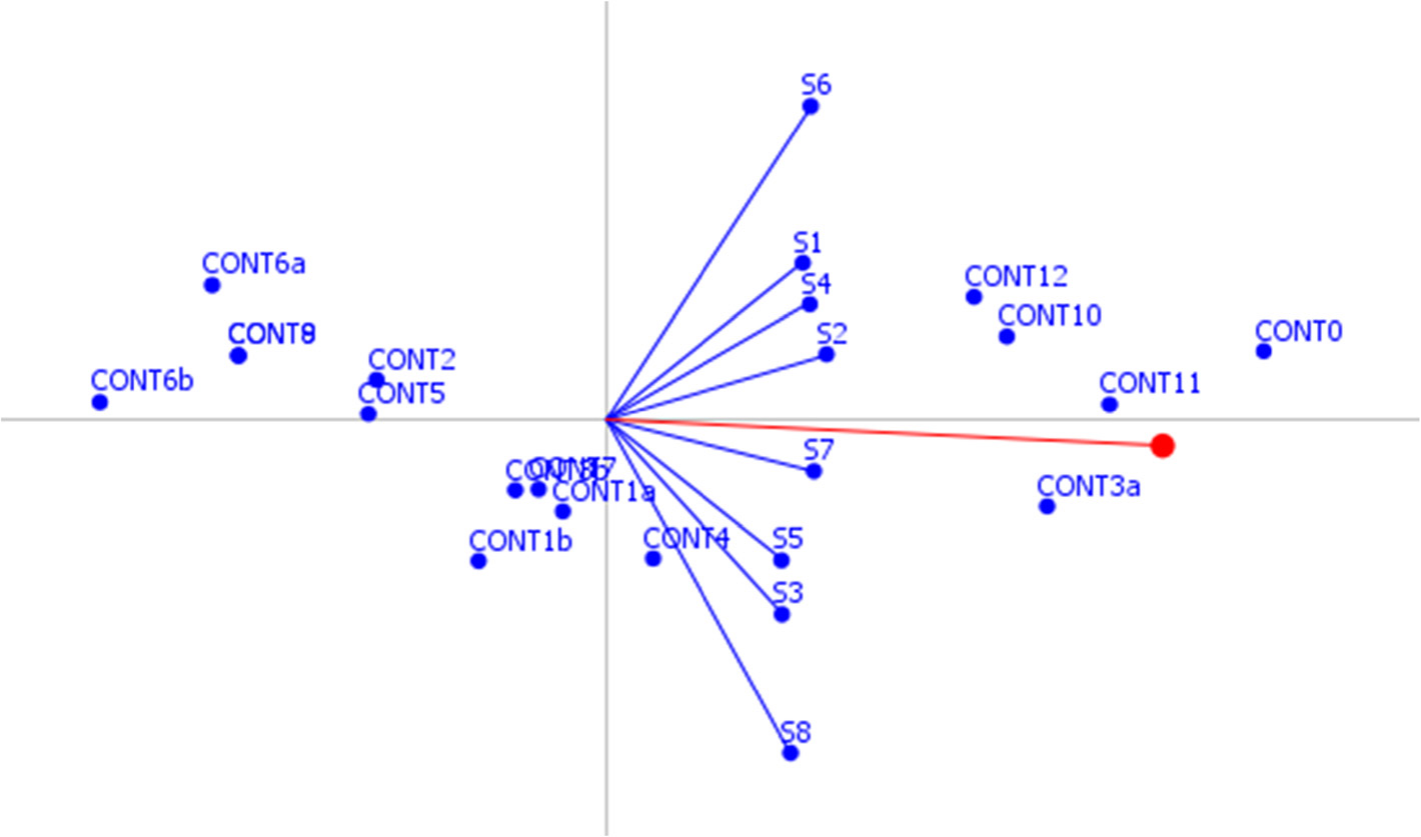
GAIA decision map for CONT model under the emergence scenario (Delta = 96,6%, meaning that 96,6% of the information is conserved in the two-dimensional representation of this map).

The GAIA plan decision map presented in Figure 2 shows the relative position of stakeholders with respect to the decision axis (in red) and selected interventions for the control model (CONT) under the “emergence scenario”. Stakeholder’s positions (represented by S1-8 in this figure) are determined by the combination of the intrinsic performance of interventions and by the stakeholder’s weighting scheme. The closer a stakeholder is to the decision axis (for example S2 and S7 in this map), the more important their agreement with the best consensual interventions (i.e. first ranked interventions), and vice-versa. Interventions located close to the decision axis (CONT0, CONT3a and CONT11 in Figure 2) are the preferred interventions for the group, and correspond to the three best ranked interventions in Table 5. A long decision axis indicates strong decision choice power whereas a short decision axis indicates that the compromise solution is close to the origin [42]. The worst ranked interventions are diametrically opposed to this axis (CONT6b, CONT6a, and CONT8). In Figure 2, all eight stakeholders point toward the right of the x axis, meaning that there are no major discordances in their preferences. Stakeholder 6 and stakeholder 8 are located somewhat apart from the rest of the group’s preferences and had the most different preferences and individual rankings. When examining their individual rankings and intervention scores (Table 6), their top three ranked interventions differ slightly from those of the group ranking even though the top three ranked group interventions are among stakeholder 6 and 8’s four highest ranked interventions (S8 even shares the same top three interventions as the group, but in a different order). This information can be used to help identify the stakeholders with distinct positions relative to the rest of the group, and represents a transparent articulation of decision considerations that need to be further discussed to reach a better consensus.

**Table 6:** Individual scores and ranking of interventions under the emergence scenario in the control (CONT) model for two stakeholders showing distinctive positions in the GAIA decision map (stakeholder 6 (S6) and stakeholder 8 (S8))

**Intervention**	**Group rank**	**Score S6**	**Rank S6**	**Score S8**	**Rank S8**
CONT0	1	0.53	1	0.3	3
CONT11	2	0.33	3	0.32	2
CONT3a	3	0.24	5	0.37	1
CONT10	5	0.37	2	0.15	5
CONT12	6	0.29	4	0.07	7
CONT4	8	−0.15	9	0.19	4
CONT1a	9	−0.11	8	0.09	6
CONT7	9	−0.18	11	0.02	9
CONT3b	10	−0.06	7	0.05	8
CONT1b	11	−0.22	12	0.02	9
CONT2	12	−0.04	6	−0.18	11
CONT5	13	−0.17	10	−0.12	10
CONT8	14	−0.26	13	−0.3	13
CONT9	14	−0.26	13	−0.3	13
CONT6a	15	−0.04	6	−0.37	14
CONT6b	16	−0.27	14	−0.29	12

**Figure 3:**
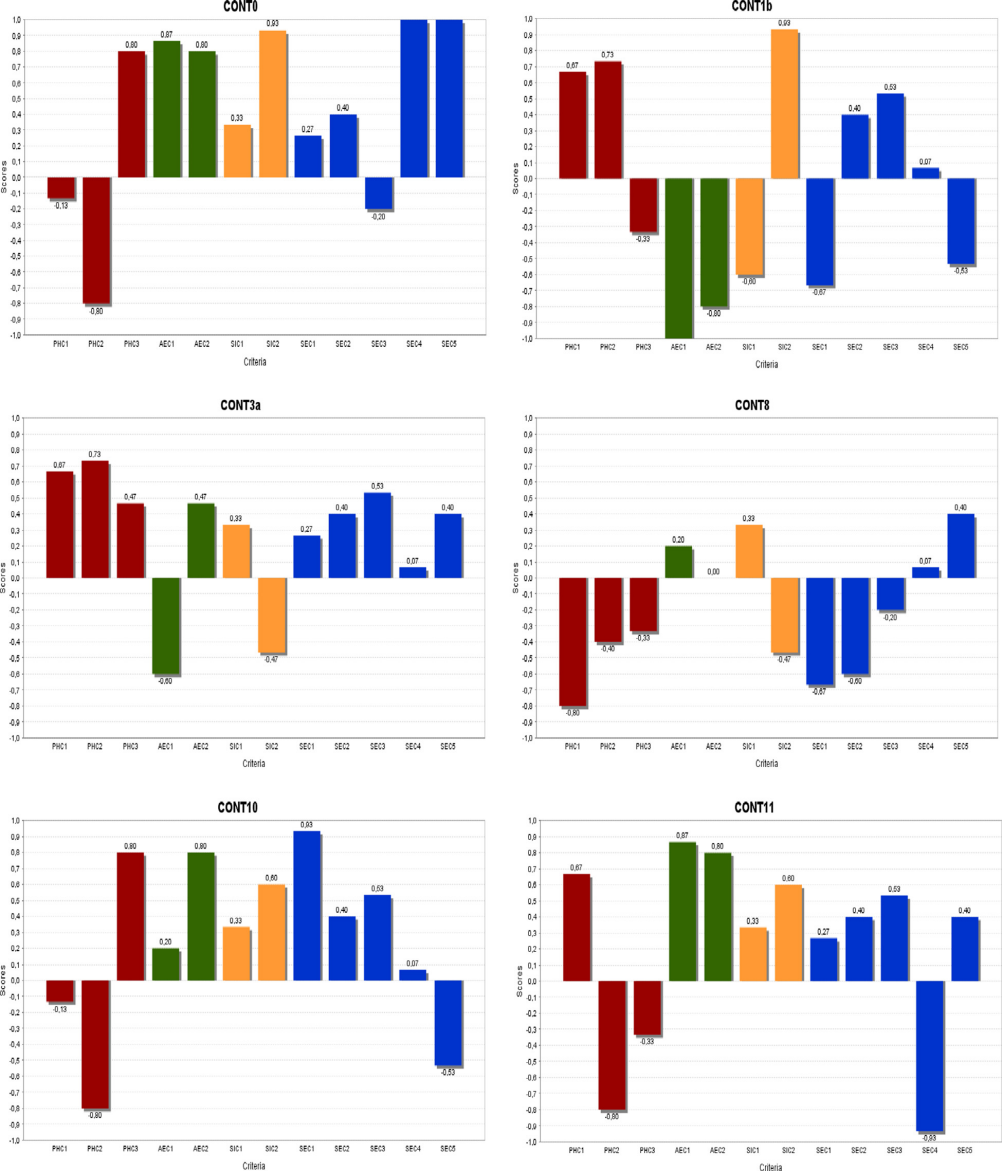
Six intervention profiles for the control (CONT) model under the “emergence scenario” (red: Public health criteria; green: Environmental and animal health criteria; yellow: Social impact criteria; Blue: Strategic, economic and operational impact criteria).

Intervention profiles graphically represent the relative intrinsic performance of an intervention on each criterion, independently of stakeholder expressed preferences (weighting schemes) (Figure 3). A descriptive analysis of the intervention profiles allows for a better understanding of the trade-off to be considered in the overall decision and to identify complementary interventions. The Y axis represents the score of the intervention for each criterion. The first ranked intervention of the group, CONT0 (status quo - basic preventive communications) performs well on animal and environmental health criteria and most operational criteria, but performs poorly on the first two Public health criteria, PHC1 (reduction of human case incidence) and PHC2 (reduction of entomological risk). Conversely, the third ranked intervention, CONT3a (small scale landscaping), performs poorly on the AEC1 criterion (impact on habitat) and SIC2 criterion (proportion of the population benefiting from the intervention), but performs more efficiently on the PHC1 and PHC2 criteria. A score of 1 on a criterion (e.g. Criteria SEC4 (Complexity) and SEC5 (Impact on organisation’s credibility) for CONT0) means that the intervention has the best intrinsic performance for this criterion among all interventions. By examining an intervention’s profile, stakeholders can consider various portfolios of interventions in order to create balanced control programmes capable of addressing multiple objectives.

A sensitivity analysis was performed to assess the impact of a stakeholder’s weighting preferences on their individual and group rankings. This analysis gives indications of the robustness of the results. Stability intervals can be generated for each stakeholder for all criteria. For example, as shown in Table 7, stakeholder 8 can change the weight given to the SEC2 criteria (cost for the private sector) from 0 to 100% without this change affecting the nature of the stakeholder’s top three ranked interventions. However, any variations in the weight of the “PHC1-Reduction in human cases incidence” criterion beyond the range of 27.5 to 38.2 will result in a change in nature of the top ranked interventions for stakeholder 3 indicating that the results are more sensitive to this later criterion.

**Table 7:** Example of a sensitivity analysis using stakeholder 8 (S8) weightings

**Criteria**	**Minimum weight**	**Actual weight**	**Maximum weight**
PHC1	27.5	30	38.2
PHC2	7.4	10	15.8
PHC3	4.9	10	12.1
AEC1	0	10	12.6
AEC2	2	10	20.2
SIC1	0	10.5	33.4
SIC2	0	4.5	8.2
SEC1	0	3	20.1
SEC2	0	3	100
SEC3	0	1.5	17.6
SEC4	0	3.8	5.1
SEC5	0	3.8	8

## Discussion

This study demonstrated the application of a transparent decision analysis method to identify decision criteria and rank interventions for the control of Lyme disease in Quebec as an illustration of its potential for the planning and management of other complex public health issues. To our knowledge, this is the first report of the use of MCDA models to document stakeholder engagement in the prioritization of Lyme disease management interventions, and the first use of such an approach to analyse public health interventions for an emerging zoonosis tackling surveillance and control interventions in a comprehensive manner. One of the key observations resulting from this study pertains to the list of criteria identified for use in the different fields of interventions relating to Lyme disease management. The general criteria categories demonstrate the comprehensiveness of the models and lend support to the use of MCDA methods as concrete applications of a “One Health” approach to zoonoses management. The list of criteria could likely be generalized or adapted for use with other vector-borne and zoonotic disease management problems. As an example, four of the general categories (public health, animal and environmental health, social impacts and strategic, economic and operational impacts) and several criteria identified with a different group of stakeholders for two other MCDA models developed to address communication strategies for Lyme disease in Quebec by the research team were identical to those identified for SURV and CONT models (MCDA-Lyme Consortium, 2012, data not shown). Furthermore, the criteria identified in this study correspond directly to the dimensions which should be integrated when analysing public policies, as identified and recommended by the Canadian National Collaborating Centre for Healthy Public Policy. This centre recently published a method for synthesizing knowledge about public policies, integrating six dimensions [43], which can be directly correlated to our list of criteria as shown in Table 8. This observation reinforces the fact that MCDA methods have the potential to improve public health decisions in line with the most recent national recommendations for evaluating public policies.

**Table 8:** Alignment of Canadian National Collaboration Centre for Health Public Policy dimensions with criteria identified in the study

**Canadian National Collaborating Centre for Healthy Public Policy**	**Study**	
**Dimensions**	**Criteria**	
Effectiveness	PHC1	Reduction in incidence of human cases
	PHC2	Reduction in entomological risk
	SUC1	Detection of zones where tick populations are present
	SUC2	Identification of zones where tick populations are established
	SUC3	Identification of Lyme endemic zones
	SUC4	Quality of data
Unintended effects	PHC3	Impacts of adverse health effects
	AEC1	Impact on habitat
	AEC2	Impact on wildlife
Cost	SEC1	Cost to the public sector
	SEC2	Cost to the private sector
Equity	SIC2	Proportion of the population benefitting from intervention
Feasibility	SEC3	Delay before results
	SEC4	Complexity
	SEC5	Impact on organisation’s credibility
Acceptability	SIC1	Level of public acceptance

The observed variations in stakeholder’s weighting of criteria was expected and can be explained as a result of differences in values, perspectives, objectives and expertise of the participating stakeholders, which is desirable within a interdisciplinary and multi-sectoral approach. On the other hand, changing the scenario from “emergence” to “epidemic” was found to have little effect on the results, which was unexpected for the research team. Although some additional weighting was given to the public health criteria by several stakeholders during the epidemic scenario, the changes were not sufficient to produce clear differences in the overall rankings of interventions between scenarios. More weight for public health criteria and social acceptability criteria during the epidemic scenario were expected but not clearly observed in this study. One hypothesis is that the description given of the epidemic scenario was not sufficiently alarming to prompt important differences in stakeholder weighting schemes between scenarios. Further analyses are needed under real epidemic situations to validate these observations and to correlate the findings of the MCDA analysis with actions that would actually be implemented by stakeholders in such situations.

In both models, performance evaluations of interventions used qualitative categorical indicators for every criterion. If ordinal scales were better suited for the evaluation of certain criteria, as was the case for the SEC4 (complexity) criterion, numeric scales were used to incorporate more precision for other criteria, such as PHC1 (reduction in human case incidence), SEC1 and SEC2 (costs for the public and the private sectors). Moreover, experts who contributed to the evaluation of certain parameters were also stakeholders on the MCDA process for the CONT model. If this approach can be interesting for the appropriation of the decision-making process by the stakeholders, it could also introduce bias in the evaluation of parameters, as preferences and expertise can vary among them. This reflected the data quality which was available at the time of the study and is the main limit of this study. Lyme disease is emerging in Quebec, and very few field studies have been performed to date. The data quality evaluation performed in the models was useful for identifying gaps in the scientific knowledge on public health intervention efficacy and impacts in the Quebec context. For the vast majority of interventions and criteria, expert opinions were the most effective way to obtain the data required to complete the models. More time and resources could be used to counter this weakness: field studies can be realized with the objective of increasing the precision of performance evaluations in models. For example, to assess the social acceptability of different control interventions, a Delphi survey was conducted among the stakeholders. A more representative survey conducted among the target population could increase the precision of this performance measure. Furthermore, additional analysis using the same MCDA model structure in other geographical areas where Lyme disease epidemiology is better characterised would provide an opportunity to validate the present model.

The first position occupied by CONT0 (status quo-basic preventive communications) in the group ranking for the CONT model was expected. The current communication method in place involves the diffusion of general information via the public organisation’s web interface. This intervention is a baseline intervention upon which stakeholders should build (but not eliminate) and include additional interventions to improve Lyme management.

For the SURV model, the top two ranked interventions were SURV2a (active surveillance of *I. scapularis* by flagging or dragging) and SURV2b (active surveillance of *I. scapularis* by trapping of small rodents). Again, this was expected, because these two interventions are considered the gold standard approach for Lyme vector surveillance in Canada at the moment [31]. The rank order of other SURV interventions should be interpreted relative to these gold standards in the group ranking.

Despite small differences, the decision map for the CONT model showed a good level of agreement between stakeholders. SURV model revealed similar observations. Even if these results represent only the views of a small group of stakeholders, greater differences could have been expected. For example, from stakeholders working for public environmental organisations, one could have expected stronger preferences for low environmental impact interventions, when compared to public health professionals. This observation demonstrates that public health protection is still a strong commonly held value, despite expertise and sectoral differences between stakeholders. In this study, there were no representatives from the general public or from public interest groups. This was a methodological choice made after discussion with participating stakeholders and in light of the exploratory nature of the objectives. The inclusion of such groups, either as representatives of the general public or of representatives of select interest groups (for example members of the Canadian Lyme disease foundation, CanLyme, or public park users) could help diversify the range of expressed preferences and concerns, an important dimension that should be considered in public health decision making.

Analysis of the intervention profiles allowed identification of complementary interventions for all models. The strengths and weaknesses of an intervention can be easily identified with these types of analyses, and highlights the need to include diverse interventions for an efficient prevention programme capable of responding to multiple objectives.

Sensitivity analyses conducted were limited to identifying stability intervals for weights given to criteria by each stakeholder. Other types of variation, for example on performance parameters of interventions, have to be simulated manually by changing parameters in the matrix. This was not realised in a systematic manner for this study but would be an interesting further step, particularly on parameters which are not supported by the scientific literature. This could help to identify which would be the more urgent knowledge gaps to tackle to consolidate decision-making in this context. Except for sensitivity analysis, the evaluation of a MCDA model’s validity is rarely mentioned in the scientific literature [44]. A framework for MCDA model validity testing would be of great use for public health research. This framework could include more extensive sensitivity analysis on weights and parameters, as well as a standardised approach to assess how accurately the model captures issues of importance to decision-makers.

Building on this study’s experience, the authors believe that MCDA approaches offer a structured and systematic process for identifying gaps in the scientific knowledge relating to important decision issues, and can be of great use to guide research priorities in public health in a context of finite and sometimes scarce resources. Another major advantage of formal decision analysis methods is the long-term utility it can confer to decision-makers. Once the matrix is completed (i.e. criteria, interventions and performances have been identified), stakeholders can re-evaluate their weights on each criteria as the epidemiological situation of the disease changes, and can observe the impacts on intervention prioritization. Used in this manner, MCDA models could be adapted and potentially used with real-time decision-making methods.

Some challenges arose during the study. First, depending on the scope and depth of the analysis, the MCDA process can be time-consuming: given the participatory approach used in this study, a significant time commitment was needed for meetings and discussions. Experts recommend a minimum of six months to complete an MCDA process with participative components. However, in this case, two years were needed to put together the research team and complete the study. Public health experts had no experience with MCDA methods, and the learning process demanded time and resources. This is not surprising and prolonged timelines have been observed in other multidisciplinary teams [7]. As such, MCDA approaches may be best suited for strategic public health planning rather than for daily decisions and emergency situations, unless MCDA models are already developed. Creating weighting schemes was also challenging and not intuitive for the majority of stakeholders. Individual support was needed in several cases, and participants needed a good understanding of the MCDA process to perform the weighting. Methods exist to facilitate the weighting process in MCDA approaches, such as the use of card sets to help order criteria before attributing numerical values [45]. We strongly recommend using them to increase the validity of analysis.

The participative approach used in identifying key stakeholders, criteria and potential interventions contributed to building a comprehensive structure of the key issues in need of consideration and helped capture the complexity of the health problem. The use of a participatory approach in this study also had an interesting and important consequence: the learning process put stakeholders from different sectors and organisations together and enabled mutual learning about each other’s organisation’s issues and priorities. During this project, stakeholders met between two to five times within the year to complete the process. We believe that this approach reinforces an interdisciplinary and multi-sectoral approach to public health management, leading to institutional empowerment. These long-term outcomes were previously observed in several examples of participatory approaches in health research [46].

## Conclusions

This study presented the results of an MCDA approach for the management of a complex public health issue - an emerging zoonosis - using a study of Lyme disease management in Quebec, Canada. Results showed that despite different weighting schemes among stakeholders, both models revealed a good level of agreement with regards to preferred interventions for surveillance and control of Lyme disease. The reliance primarily on expert opinion for development of the performance matrices underscores the need to enhance scientific knowledge on issues of importance for decision-making for Lyme management in Quebec. Overall, explicit decision analysis methods present themselves as an interesting systematic approach for the management of complex public health issues and particularly for emerging infectious diseases arising from human populations interacting with animals and the environment. Future steps should include an assessment of the applicability and usefulness of the proposed models for program-level decision support pertinent to the prevention and control of Lyme disease in Canada. Moreover, building on the outcome of this project, future projects should evaluate the utility of MCDA for structuring a formal decision analysis approach for other complex public health issues (e.g.: prevention of foodborne and waterborne diseases, antimicrobial resistance, influenza, and environmental health issues) as well as within various ongoing processes and prioritization settings targeting effective multi-sectoral engagement. The development of a methodological framework for MCDA model validation would also be of great interest for the public health sector.

## Abbreviations

AEC: Animal and environmental health criteria; COMM: Communication model; CONT: Control model/Control interventions; SOC: Strategic, economic and operational criteria; HSSA: Health and Social Services Agency; MCDA: Multi-criteria decision analysis; PHC: Public health criteria; SIC: Social impact criteria; SUC: Surveillance criteria; SURV: Surveillance model/Surveillance interventions.

## Competing interests

The authors declare that they have no competing interests.

## Authors’ contributions

JPW, DB and PM directed the study. All authors contributed to the study design. AGH, CA, KS, VH played lead roles in the data collection process. Data analysis was performed by CA, HDC, KS and VH with guidance from other authors. CA and VH wrote the first draft of the manuscript. JPW, DB, PM, AGH and HDC contributed to further drafts. All authors read and approved the final manuscript.

## Pre-publication history

The pre-publication history for this paper can be accessed here:

http://www.biomedcentral.com/1471-2458/13/897/prepub
